# The nonlinear motion of cells subject to external forces†

**DOI:** 10.1039/d2sm00934j

**Published:** 2022-11-10

**Authors:** Aondoyima Ioratim-Uba, Aurore Loisy, Silke Henkes, Tanniemola B. Liverpool

**Affiliations:** a School of Mathematics, University of Bristol Bristol BS8 1UG UK deems.ioratim-uba@bristol.ac.uk; b Lorentz Institute for Theoretical Physics, Leiden University Leiden 2333 CA The Netherlands

## Abstract

To develop a minimal model for a cell moving in a crowded environment such as in tissue, we investigate the response of a liquid drop of active matter moving on a flat rigid substrate to forces applied at its boundaries. We consider two different self-propulsion mechanisms, active stresses and treadmilling polymerisation, and we investigate how the active drop motion is altered by these surface forces. We find a highly non-linear response to forces that we characterise using drop velocity, drop shape, and the traction between the drop and the substrate. Each self-propulsion mechanism gives rise to two main modes of motion: a long thin drop with zero traction in the bulk, mostly occurring under strong stretching forces, and a parabolic drop with finite traction in the bulk, mostly occurring under strong squeezing forces. In each case there is a sharp transition between parabolic, and long thin drops as a function of the applied forces and indications of drop break-up where large forces stretch the drop.

## Abstract 2

To develop a minimal model for a cell moving in a crowded environment such as in tissue, we investigate the response of a liquid drop of active matter moving on a flat rigid substrate to forces applied at its boundaries.

## Graphical abstract

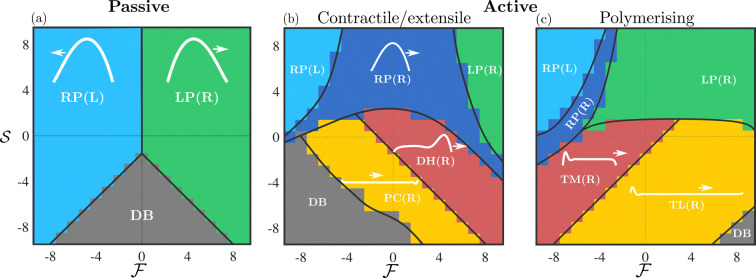

## 1 Introduction

Cells are highly adaptable and move themselves around in a variety of different conditions and environments.^1–3^ This is essential for biological functions such as wound repair,^4^ organ development,^5^ and in pathological processes such as cancer metastasis.^6^ Understanding individual cell motility and how it affects collective cell migration is key to understanding these processes. In particular, experiments show that cell–cell tugging plays an important role in collective migration,^7–9^ and that the force distribution within tissues may tell us something about pathological behaviour.^10,11^

Cell motility is powered by the cytoskeleton, a dynamic network of interlinking protein filaments inside the cell.^12–14^ These filaments can collectively form anisotropic liquid crystalline (LC) phases.^15^ There are a number of mechanisms by which cell motility occurs.

The most studied is cell crawling,^16–21^ which combines the treadmilling (polymerisation/depolymerisation) of cytoskeletal actin filaments with strong adhesion to the substrate. In cells the likely source of this type of motion is actin polymerisation combined with acto-myosin contractility. Myosin II molecular motors cause actin filaments to slide relative to each other^22,23^ and generate an active stress that can be contractile (positive) or extensile (negative). A large class of hydrodynamic active liquid models of these processes have been built, using active gel theory,^24–28^ and also more detailed computational active nematic LC models.^29^ Such models can be augmented by including reaction-diffusion chemical feedback,^30,31^ and by adding confinement.^32,33^ It has been shown that even in the absence of actin treadmilling, spontaneous motion is still possible in LC active matter systems.^34–38^

A minimal description of cell motility is thus provided by the motion of a drop of anisotropic active LC matter. The possible modes of a such a drop freely moving on a flat rigid substrate have recently been classified by some of us in ref. 39. We identified three modes: motion due to active stresses, motion due to self-advection of active units along their direction of orientation, and motion due to contact angle mismatch. All modes are in principle present but one can consider regimes when one mode dominates. We also showed that a drop moving purely due to active stresses can do so without exerting traction on the substrate.^40^ This type of motion is relevant to fast migration in crowded environments,^2,41^ where cellular adhesions are unstable at high strain rates.^42^

Crowded environments also lead to significant forces on cells. However, many of the effects of external forces on cell-motility and migration remain a mystery. These forces, either coming from cell–ell tugging or from outside the cell, are important for the functionality of cells and tissues. Furthermore, experiments show that external forces can alter cell stiffness, induce migration, alter cell shape, induce remodelling, and alter cell phenotype.^43–45^ Hence a better handle on them promises to have a significant impact on our understanding of multicellular systems.

In this paper we study the dynamics of an active LC drop on a flat surface under external forces applied at its two ends. We model the anisotropy of the cytoskeleton in two ways, corresponding to dynamics dominated by the first two modes we identified in ref. 39. First, we probe an imposed LC director field, and the active stress that this generates (active contractile/extensile drop). The behaviour is controlled by the ratio 
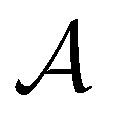
 of the activity to the splay-bend winding number of the LC director, as measured from the surface to the top of the drop. Second, we study self-advection of LC units along their direction of orientation (active polymerising drop), whose behaviour is controlled by the ratio 
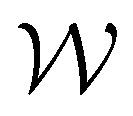
 of the self-advection speed to the surface tension. We classify the motion of the drop according to (1) the difference between the forces on each end, *i.e.* whether it is being squeezed or stretched and (2) the sum of the forces applied to its two ends, *i.e.* if it is being pushed to the right (R) or to the left (L). For a passive drop (
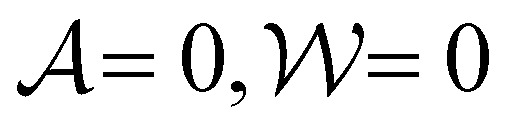
), we find parabolic shapes with simple symmetric behaviour: it moves to the right (or left) if it is pushed in the right (or left) direction unless it is stretched above a critical stretching force where it tends to break up into smaller droplets. For both active drops, we find a much richer response to external forces. The active contractile/extensile drop (
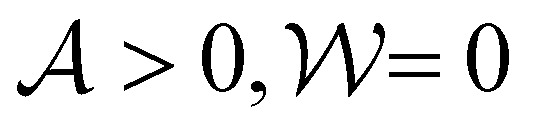
) drop moves to the R almost all the time except when the sum of forces is large in the L direction and it is being squeezed. We also find a wide variety of shapes. Parabolic shapes are observed only when the drop is being squeezed. When the drop is being stretched, we find (i) a double humped shape with a large hump at the front when the sum of forces is strongly to the R, (ii) a flat pancake shape which exerts almost no traction on the surface when the sum of forces is small and (iii) droplet break up when the sum of forces is strongly in the L direction. Our results hold for both contractile and extensile drops as our equations of motion are invariant: changing the sign of 
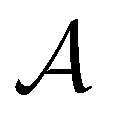
, which flips the directions L ↔ R, is equivalent to either moving from contractile to extensile stresses or to flipping the splay-bend winding number. The active polymerising drop (
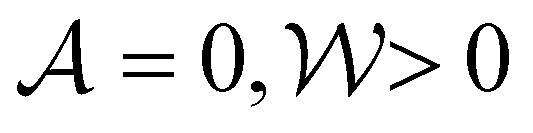
) also moves to the R almost all the time, except when the sum of forces is large in the L direction and it is being squeezed. Again, parabolic drops are observed only when the drop is being squeezed. When the drop is being stretched, we find (i) a double humped shape with a large hump at the rear when the sum of forces is strongly to the L, (ii) a flat travelator shape which exerts almost no traction on the surface and (iii) droplet break up when the sum of forces is strongly in the R direction. Changing the sign of 
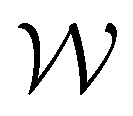
 flips the directions L ↔ R.

## 2 Model

**Fig. 1:**
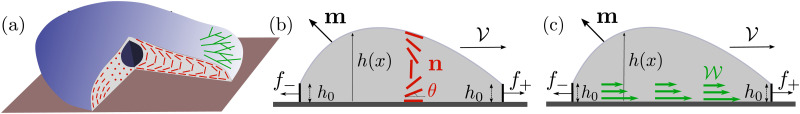
(a) Cartoon of a crawling cell with slice removed. The configuration of the actin filaments is shown in red, and polymerising filaments are shown in green. (b) Schematic showing a contractile/extensile drop on a flat surface being pushed/pulled at its boundaries by external forces *f*_±_ (which can also stretch or squeeze the drop), positive *f*_+_ or *f*_−_ means that the force is directed to the right. The nematic director is denoted by **n**. (c) Schematic showing a polymerising drop with external forces *f*_±_. In both cases, the boundaries are set to be at a height *h*_0_ above the solid substrate (*z* = 0).

We model a single cell as a two dimensional incompressible active nematic drop moving on a flat rigid substrate subject to external forces at its boundaries (Fig. 1(a)). This can be thought of as a projection of the full 3d system to its average in 2d. We expect this minimal model to capture a large part of the phenomenology in 3d but not the full behaviour such as splay in the lamellipodial protrusion.^29^ We work in the (*x̃*,*z̃*) plane, where the drop is characterised by the height *h̃*(*x̃*,*t̃*) of its free surface above the substrate, and moves with velocity 
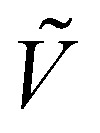
. Note that variables with tildes are dimension-full, while the un-tilded variables, which we introduce for the lubrication approximation,^51^ are dimension-less. We use the well established equations of active liquid crystal hydrodynamics^46,47^ where the motion of the coarse grained orientation of elongated units **n** = (cos *θ*,sin *θ*), known as the director, is coupled to the fluid velocity **ũ** inside the drop. The velocity satisfies force balance equations at vanishing Reynolds number:

1a ∂_*j*_*

<svg xmlns="http://www.w3.org/2000/svg" version="1.0" width="16.000000pt" height="16.000000pt" viewBox="0 0 16.000000 16.000000" preserveAspectRatio="xMidYMid meet"><metadata>
Created by potrace 1.16, written by Peter Selinger 2001-2019
</metadata><g transform="translate(1.000000,15.000000) scale(0.015909,-0.015909)" fill="currentColor" stroke="none"><path d="M400 840 l0 -40 -40 0 -40 0 0 -40 0 -40 40 0 40 0 0 40 0 40 80 0 80 0 0 -40 0 -40 80 0 80 0 0 40 0 40 40 0 40 0 0 40 0 40 -40 0 -40 0 0 -40 0 -40 -80 0 -80 0 0 40 0 40 -80 0 -80 0 0 -40z M320 520 l0 -40 -80 0 -80 0 0 -80 0 -80 -40 0 -40 0 0 -120 0 -120 80 0 80 0 0 -40 0 -40 160 0 160 0 0 40 0 40 40 0 40 0 0 200 0 200 80 0 80 0 0 40 0 40 -240 0 -240 0 0 -40z m240 -160 l0 -120 -40 0 -40 0 0 -80 0 -80 -80 0 -80 0 0 40 0 40 -40 0 -40 0 0 120 0 120 80 0 80 0 0 40 0 40 80 0 80 0 0 -120z"/></g></svg>

*_*ij*_ + *f̃*_*i*_ = 0,

1b **_*ij*_ = −*p̃δ*_*ij*_ + *η*(∂_*i*_*ũ*_*j*_+ ∂_*j*_*ũ*_*i*_) − *α̃n*_*i*_*n*_*j*_,

where **f̃** = **f̃**(*x̃*) is the external force per unit height, *p̃* is the pressure, **ũ** is the fluid velocity inside the drop, and 
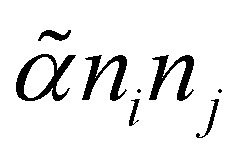
 is the active stress to leading order in a gradient expansion, which represents the coarse-grained stresses generated when cytoskeletal actin filaments slide relative to each other.^48,49^ Following,^39,40^ We perform calculations in the lubrication approximation^51^ for which changes in the height are much smaller than the width. In this approximation, higher order gradients in the stress tensor can be neglected because they scale with the ratio of the characteristic length to the characteristic height of the drop, which is small. We also work in the strong elastic limit, where the director relaxes instantaneously to follow changes in the drop height *h̃*(*x̃*,*t̃*). In this case, the dynamic equation for the director reduces to 
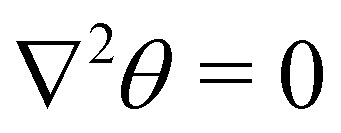
.

**Fig. 2:**
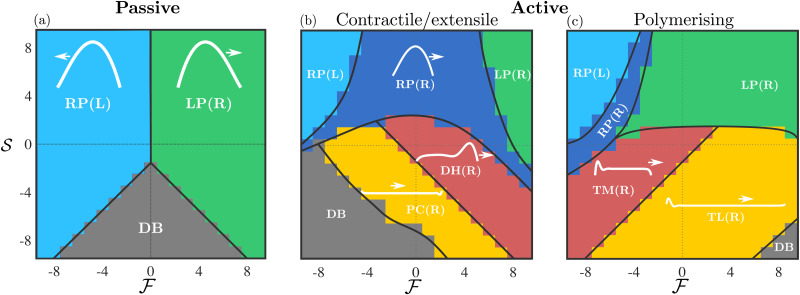
(a) Schematic phase diagram for a passive drop. The light blue region corresponds to RP(L) right-parabola left-moving drops, green corresponds to LP(R) left-parabola right-moving drops, while droplet breakup DB is grey. (b) Schematic phase diagram for a contractile/extensile drop. New phases include the RP(R) right-parabola right-moving state (dark blue), the DH(R) double-hump right-moving (coral) and the PC(R) pancake right-moving (yellow) states. (c) Schematic phase diagram for a polymerising drop. The RP(L), LP(R), and RP(R) phases are present, while the DH(R) double-hump, right-moving is replaced by the TM(R) tread-milling drop right-moving (coral), and the PC(R) pancake right-moving is replaced by the TL(R) travelator right-moving (yellow). Characteristic drop shapes are superimposed in white.

For the active contractile/extensile drop, we anchor the director parallel to the substrate, *i.e. θ*(*z̃* = 0) = 0, and parallel to the free surface with an imposed winding, *i.e. θ*(*z̃* = *h̃*) = *ω*π+ arctan(*h*′). The winding number *ω* ∈ *ℤ*^+^ counts the number of half turns of the director across the drop height (Fig. 2). At fixed activity, the winding of the director, through its chirality, breaks the left-right symmetry and sets the preferred direction of motion. With no external forces, at positive activity (extensile drop), the drop moves right for positive winding and left for negative winding. There is no active contribution to the drop velocity when *ω* = 0. For the active polymerising drop, we anchor the director parallel to the substrate *i.e. θ*(*z̃* = 0) = 0, and parallel to the free surface with no imposed winding, *i.e. θ*(*z̃* = *h̃*) = arctan(*h*′).

**Fig. 3:**
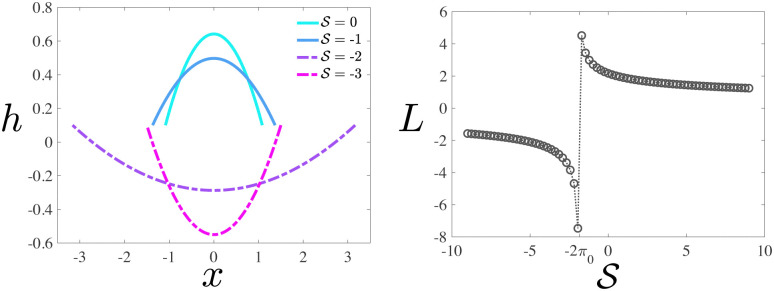
Left: Droplet height profiles at 
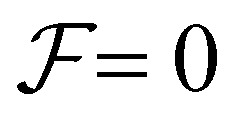
 and 
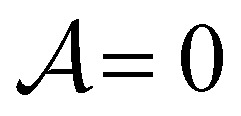
. Here, π_0_ = 0.9226 and *Ω* = 1. Right: Drop length *L* as a function of 
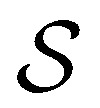
.

The external forces 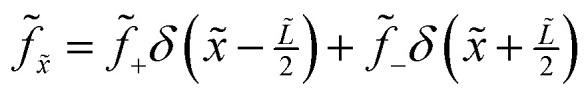, *f̃*_*z̃*_ = 0 are localised at the left and right drop borders, which leads to boundary conditions on the pressure 
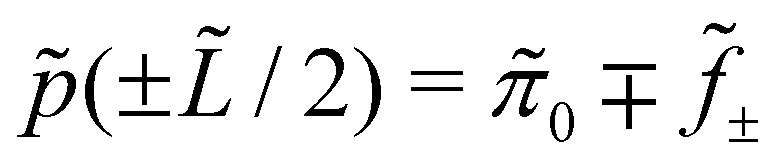
, and 
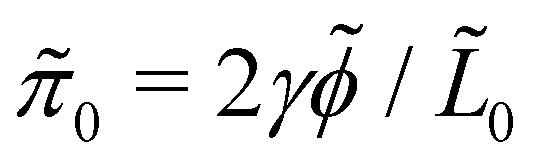
 is the Laplace pressure generated by the surface tension *γ* of an un-forced symmetric passive drop of length *L̃*_0_ and contact angle *φ̃*. For an active drop with no external forces, 
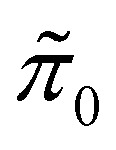
 is a constant shift in the pressure. These boundary conditions can be derived by integrating the *x̃* component of (1a) with respect to *x̃* from *L̃*/2 − *Δ* to *L̃*/2 + *Δ* and from *−L̃*/2 − *Δ* to *−L̃*/2 + *Δ*, where *L̃* is the length of an arbitrary drop with *Δ* > 0, and taking the limit *Δ* → 0^+^ (see Section 1.2 of ESI ref. 50). In addition to these boundary conditions, we use a free surface boundary condition for the stress at the free surface: ***σ̃***·***m*** = *γκ****m*** for the stress at the free surface, where *γ* is the surface tension, *m* is the unit normal vector (see Fig. 1(a)), and *κ* = ∇·***m*** is the curvature of the free surface. The interaction of the drop with the rigid substrate is modelled by a partial slip boundary condition: *ũ*_*x̃*_ = *l̃*_*u*_*σ̃*_*x̃Z̃*_/*η*, where *l̃*_*u*_ is a slip length.

We seek travelling wave solutions *h̃* = *h̃(x̃* − *Ṽt*) and work in the reference frame of the drop. Mass conservation implies2
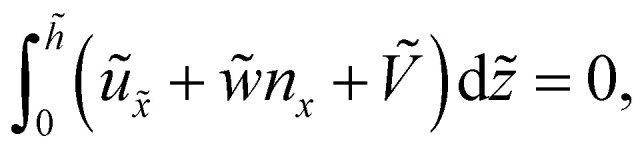
where ***ũ*** is the fluid velocity inside the drop satisfying force balance and incompressibility, and *w̃n* describes the additional transport due to treadmilling self-advection at speed *w̃* of active units whose orientations are characterised by the director *n* – the active units essentially propel themselves along their own tangent. Since treadmilling self-advection only occurs near substrates, we choose the form *w̃* = *w̃*_0_e^−*h̃*/*l̃*_*w*_^, where *w̃*_0_ is a characteristic self-advection speed and *l̃*_*w*_ is the characteristic height over which the self-advection term decays in the direction normal to the substrate. This functional form was also used in previous work that simulates crawling cells.^29^ Then, combining mass conservation with *p̃* = −*γp̃*′′, which can be derived by taking the leading order terms of the normal component of the stress boundary condition at the drop free surface, we obtain a non-linear ODE for *h̃*. After non-dimensionalisation, we retain the salient dimensionless parameters activity 
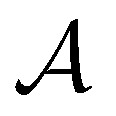
, advection speed 
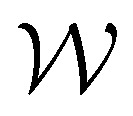
, and drop velocity 
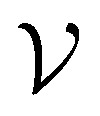
,3
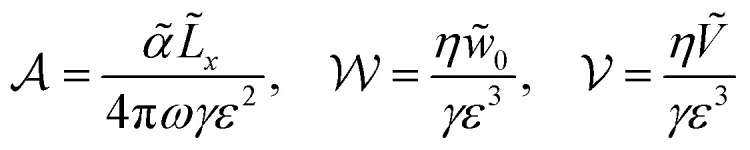
and also height *h* = *h̃*/*εL̃*_*x*_, coordinates *x* = *x̃*/*L̃*_*x*_, *z* = *z̃*/*εL̃*_*x*_, slip length *l*_*u*_ = *l̃*_*u*_/*εL̃*_*x*_, polymerisation height *l*_*w*_ = *l̃*_*w*_/*εL̃*_*x*_, pressure 
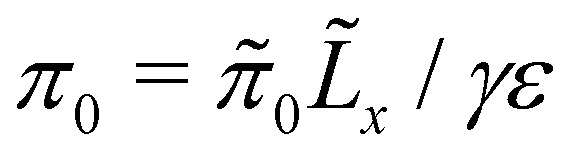
, contact angle 
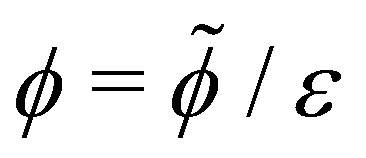
, and forces *f*_±_ = *f̃*_±_*L̃*_*x*_/*γε*, where *L̃*_*x*_ is a characteristic length scale in the *x* direction. Then the drop shape satisfies the ODE4
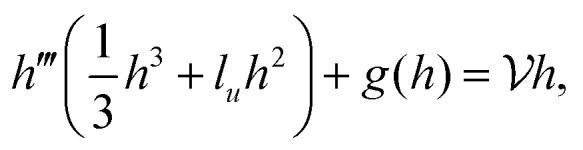
where 
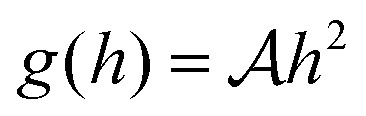
 for the contractile/extensile drop and 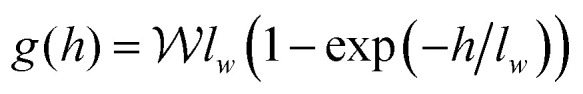 for the polymerising drop. The drop velocity 
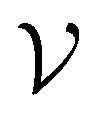
 is a functional of *h*:5
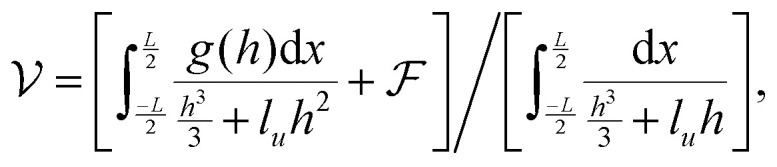
where we have introduced push 
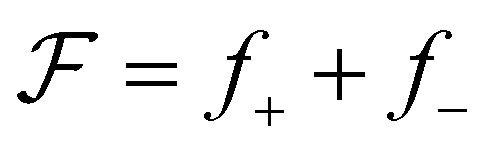
 (positive means push right and negative means push left). We also use the quantity 
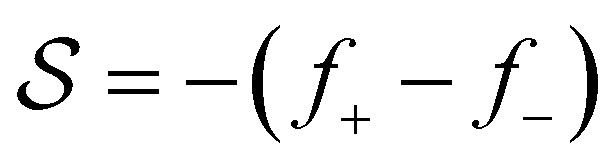
, which we term squeeze, in the analysis of our results (positive means squeeze, and negative means stretch). We set the height of the drop at its boundary to a finite *h*_0_ = *h*(±*L*/2), which represents a finite contact area with neighbouring cells or with obstacles. The drop length *L* is determined as part of the solution by requiring that the drop have constant volume *Ω*: 
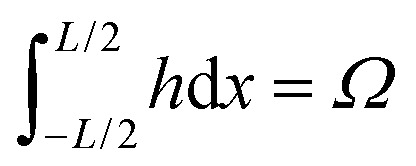
. The tangential traction exerted by the drop on the substrate is *σ*_*xz*_|_*z*= 0_ = *hh*′′′. In terms of *h* alone, using (4), the traction can be written 
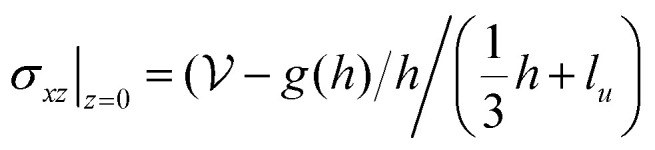
. The dimensionless parameters 
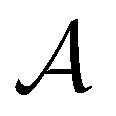
, 
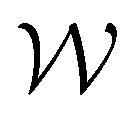
, 
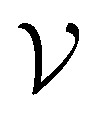
, 
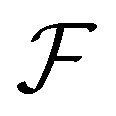
, and 
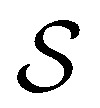
 all represent a ratio of stress to surface tension, and as we will show below, the interesting active phenomenology appears when they are all 
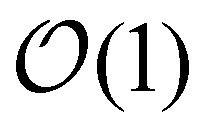
. We translate these results back to biological parameters at the end, allowing us to make predictions for cell speeds and stresses in addition to cell shapes.

### 2.1 Numerics

We obtained solutions to (4) by numerically solving the time dependent version of the force balance equation, ∂_*t*_*h*+ ∂_*x*_*I* = 0, where 
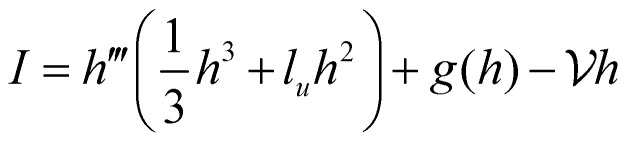
, which represents overdamped dynamics of the height evolution. Solving the time dependent problem (starting from a known initial condition) allows us to iteratively evaluate the integrals in eqn (5) and gives us the potential to investigate the temporal dynamics of the drop. The steady state of the time evolution, ∂_*t*_*h* = 0, is equivalent to eqn (4).

We used an implicit method, a variant of the Crank–Nicholson algorithm, using finite difference coefficients to approximate the derivatives. The algorithm starts with an initial condition that has the correct values of *h* at the boundaries and advances in time until steady state. The algorithm advances in time according to *h*^*n*+1^_*i*_/Δ*t* + *I*_*i*_(*h*^*n*+1^_1_,…,*h*^*n*+1^_*N*_)/2 = *h*^*n*^_*i*_/Δ*t* + *I*_*i*_(*h*^*n*^_1_,…,*h*^*n*^_*N*_)/2, where *h*^*n*^_*i*_ is the discretised drop height evaluated at the *i*th spatial point at the *n*th time step, *I*_*i*_(*h*^*n*^_1_,…,*h*^*n*^_*N*_) is the discretised version of *I* as defined above evaluated at the *i*th at the *n*th time step. The time stepping in the algorithm is adaptive, meaning that the time step is increased as steady state approaches.^39^ In the numerics, we apply both height and pressure boundary conditions (converted to boundary conditions on *h*′′) because the time evolution is a fourth order PDE:

6 *h*(±*L*/2) = *h*_0_, *h*′′(±*L*/2) = ±*f*_±_ − π_0_.

The boundary conditions on *h*′′ are required for consistency with force balance, however we are in principle free to change the boundary conditions on *h*. In all simulations *l*_*u*_ = 0.05, *h*_0_ = 0.1, *Ω* = 1, π_0_ = 0.9226, which corresponds to a contact angle of 1 for a free passive drop, *ω* = 1, and the spatial step size is 0.002. The value of the slip length was chosen to be consistent with ref. 39. Changing the slip length has different effects on the contractile/extensile mode and polymerisation mode: increasing the slip parameter at a fixed polymerisation speed decreases drop speed because motility *via* polymerisation requires strong adhesion.^29,39^ The opposite is true for the contractile/extensile drops – increasing the slip length increases drop speed.^39^

## 3 Results

**Fig. 4:**
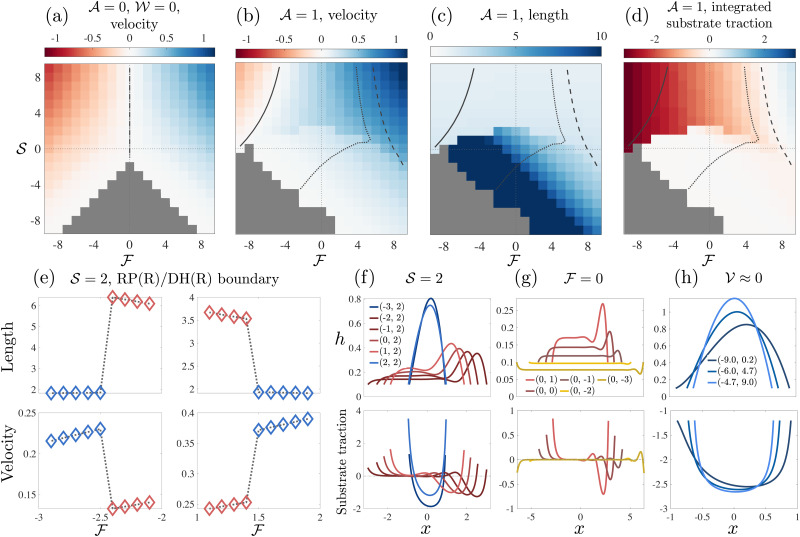
Contractile/extensile drops. (a) Velocity of a passive drop. (b) Velocity of a contractile/extensile drop. (c) Length of a contractile/extensile drop. (d) Traction between the drop and the substrate integrated over 70% of the drop length: 
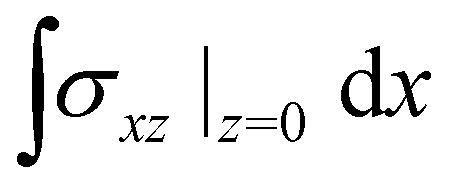
. Each square represents a single simulation and its colour corresponds to the legend. The solid line in each plot is an isoline corresponding to zero velocity, the dashed line is an isoline where the first moment of *h*, 
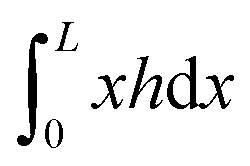
, vanishes, and the dotted line is an isoline corresponding to the drop having equal contact angles. (e) 2nd moment and velocity 
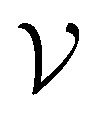
 across the RP(R)/RH(R) boundary, indicating a first order transition. (f) Stable numerical solutions for *h* (top) and the corresponding traction *σ*_*xz*_|_*z*=0_ (bottom) for 
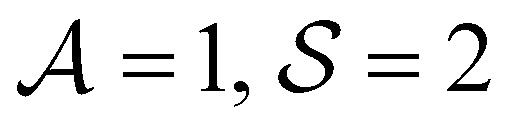
, (g) 
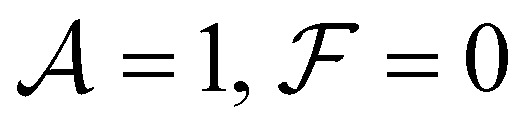
, (h) 
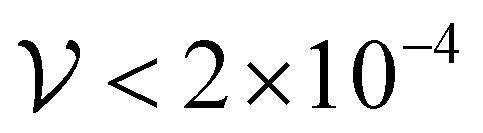
. The colour of each curve matches the colour of its phase in Fig. 2(c). The legend labels are coordinates in the (
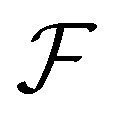
, 
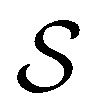
) phase plane.

**Fig. 5:**
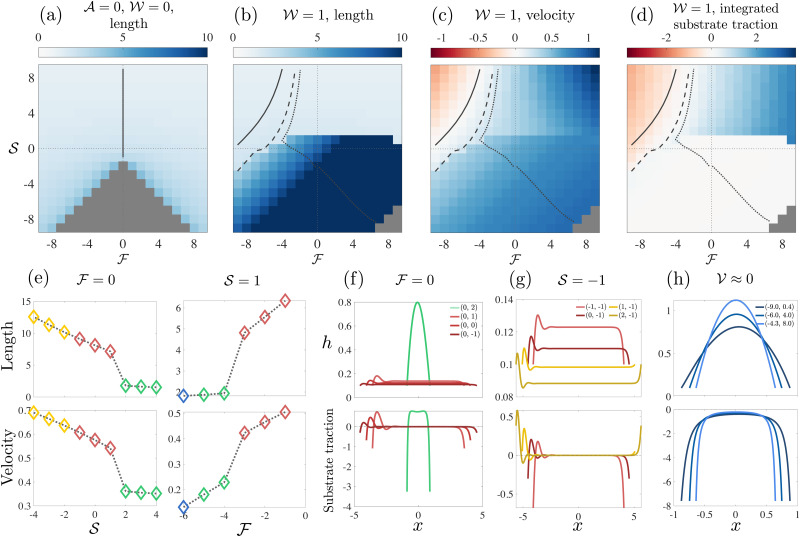
Polymerising drops. (a) Length of a passive drop. (b) Length of a polymerising drop. (c) Velocity of a polymerising drop. (d) Traction between the drop and the substrate integrated over 70% of the drop length: 
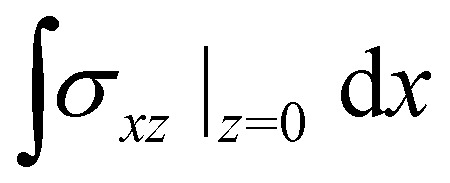
. Each square represents a single simulation and its colour corresponds to the legend. The solid line in each plot is an isoline corresponding to zero velocity, the dashed line is an isoline where the first moment of *h*, 
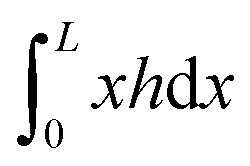
, vanishes, and the dotted line is an isoline corresponding to the drop having equal contact angles. (e) Jump in length and velocity going from parabolic to flat. (f) Stable numerical solutions for *h* (top) and the corresponding traction *σ*_*xz*_|_*z*=0_ (bottom) for 
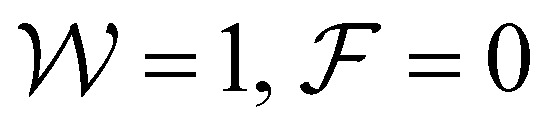
, (g) Stable numerical solutions for *h* (top) and the corresponding traction *σ*_*xz*_|_*z*=0_ (bottom) for 
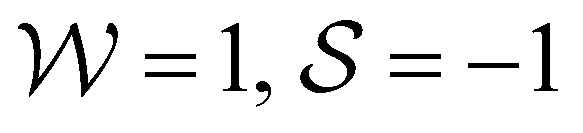
, (h) 
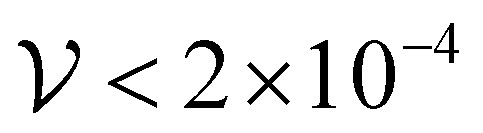
. The colour of each curve matches the colour of its phase in Fig. 2(c). The legend labels are coordinates in the (
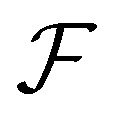
, 
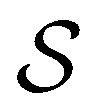
) phase plane.

The phase diagrams in Fig. 2(a)–(c) schematically show the regimes of drop shapes and motility that we find for the passive 
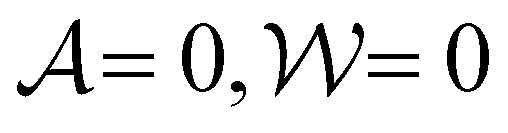
, the contractile/extensile case 
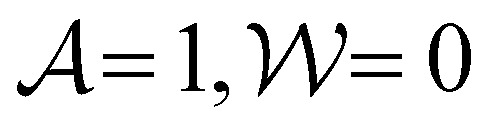
 case, and the polymerising case 
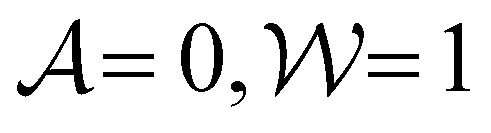
. Fig. 4 and 5 provide full results for drop velocity, drop shapes and substrate traction.

### 3.1 Passive drop

We begin with a passive drop 
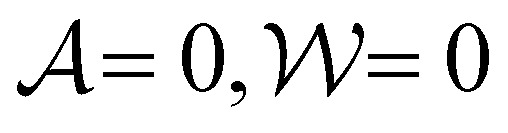
 (Fig. 2(a)), both to provide a reference, but also to illuminate the limit when surface tension dominates over activity. Then all dimensionless parameters are small and eqn (4) can be approximated by *h*′′′(*x*) = 0, which is the equation for the passive drop with a parabolic solution. Moving to finite 
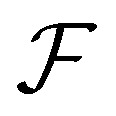
 and 
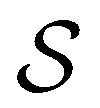
 now, we observe two phases of motion, both with finite traction in the bulk: a left-moving right-parabola RP(L) for 
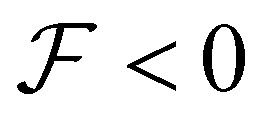
 and a right-moving left-parabola LP(R) for 
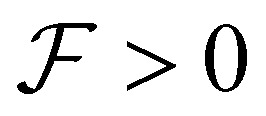
. These passive drops are qualitatively close in shape to an upside down parabola but are asymmetric with a first moment of *h* that is non-zero (characteristic shapes shown in Fig. 2(b)). Squeezing the drop 
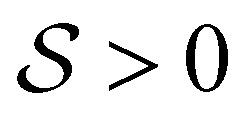
 in general maintains the parabolic shape and increases the effective surface tension.

The passive solutions are antisymmetric along the push/pull 
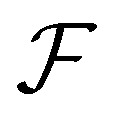
 axis, and they move with opposite velocities that increase as the drop is squeezed (see Fig. 4(a)). All drops become longer and thinner as we move along the stretch/squeeze axis from squeeze to stretch, eventually reaching the region of drop breakup (DB), where the drop free surface reaches *h* < 0. For passive drops at 
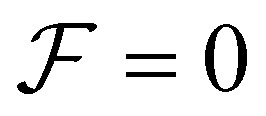
, there are no steady solutions in the DB region that satisfy volume conservation. Consider a passive drop with 
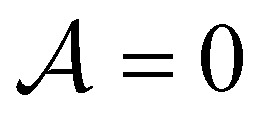
 and 
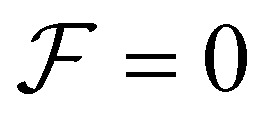
. The equation for drop height becomes7
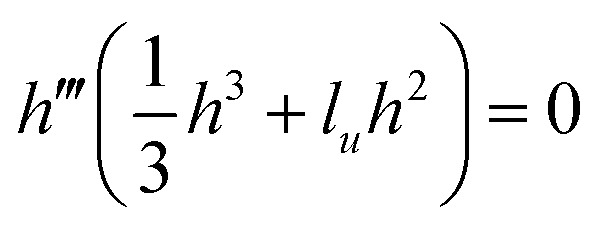
and has the solution8
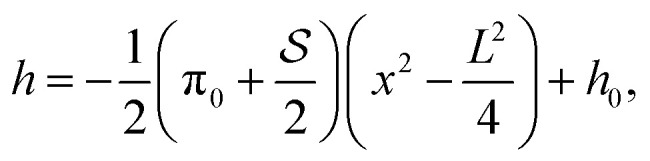
where we have used *h*(±*L*/2) = *h*_0_ and *h*′′ = *f*_+_−π_0_. The drop length *L* is determined by the volume constraint 
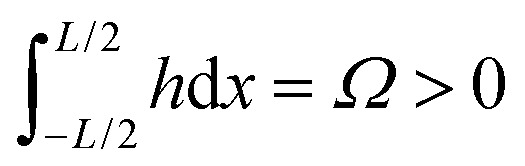
. The drop length diverges at 
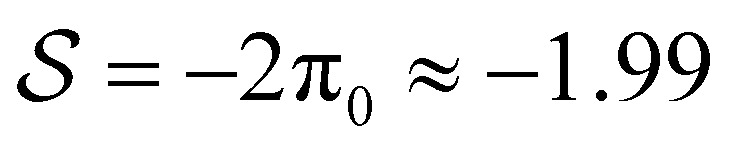
 (π_0_ is chosen so that the contact angle of a free passive drop is one), and goes negative for 
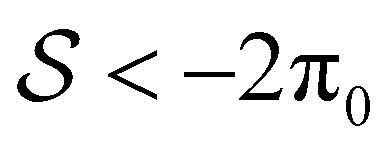
. This means that volume conservation is not truly satisfied even though the condition 
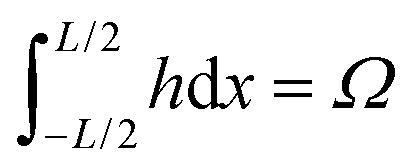
 is satisfied mathematically by a negative *L*. This is clear in the left plot in Fig. 3, where *h* < 0 for 
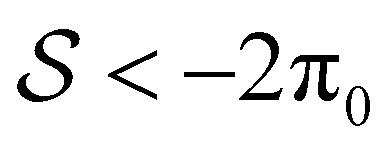
 and thus 
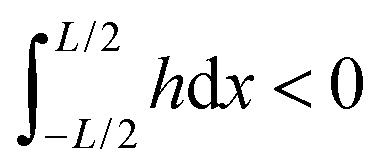
, with |*L*| being the actual length of the drop.

### 3.2 Active contractile/extensile drop

With contractility, as shown in shown in Fig. 2(b), we find, in addition to RP(L), LP(R) and DB, a right-moving right-parabola RP(R) state with finite traction, and then two long and thin states (see length in Fig. 4(c)), the right-moving double hump DH(R) state, which has a small dip followed by a prominent frontal hump, and the right-moving pancake PC(R) drop, which is flat almost everywhere with the average height *h̄* < *h*_0_.

Both DH(R) and PC(R) drops have zero traction in the bulk (Fig. 4(d)). In,^40^ equal contact angles were imposed at the boundaries for all drops (rather than variable external force), and it was found that the tractionless DH(R) shapes emerged when the dimensionless active contractile stress 
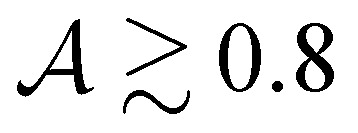
. Here, these tractionless drops can be found in the DH(R) region of the phase plane along the line of equal contact angle (dotted line, Fig. 4(b)–(d)). The drop partitions itself into a small region with large free surface curvature, and a larger region where the free surface is flat. There is large curvature at these humps, and the remaining 50–90% of the drop is flat. At lower activity 
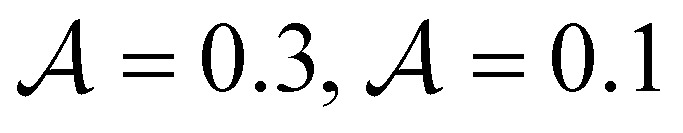
 (see ESI ref. 50, Fig. S2), we find DH(R) and PC(R) shapes at large enough stretch (
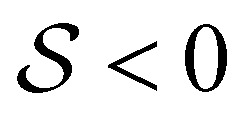
), which shows that stretching can work together with activity to deform the drop. Therefore, in the DH(R) and PC(R) drops, we see the competition between active contractile/extensile stress plus stretch, and surface tension: activity plus stretch wants to deform the drop, and surface tension wants the free surface to be flat.

The symmetry about the push/pull axis is broken because the drop is motile at zero force due to the imposed winding *ω* = 1 (Fig. 4(b)). Both the RP(L) and LP(R) regions become smaller with activity and are pushed towards the top-left (push-left/squeeze) and top-right (push-right/squeeze) of the phase plane respectively by the emergence of the RP(R) phase, which largely occupies the middle squeeze region that lies between RP(L) and LP(R). The stretch region of the phase plane is populated by DH(R) and PC(R), as well as DB. The region containing DH(R) and PC(R) shares a phase boundary with RP(R) determined by the drop length, and the distribution of substrate traction. The second moment of *h* (with drop length scaled to unity), which is a measure of the spread of the drop also determines the same phase boundary – see ESI.† The phase boundary is indicated in the schematic Fig. 2, as determined from the data in Fig. 4(c) and (d). Strikingly, the transition between RP(R) and DH(R)/PC(R) is sharp suggesting a first order transition.

The behaviour of the second moment and the drop velocity across the phase boundary is shown in Fig. 4(e). There is a jump in both quantities going from RP(R) to DH(R) and also from DH(R) to RP(R). The change in drop shape induced by moving from RP(R) to DH(R) and back to RP(R) at constant squeeze 
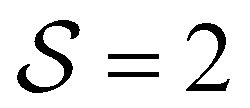
 is shown if Fig. 4(f). The drop shape changes continuously within the DH(R) region but changes sharply at the phase boundary. There are also indications of bi-stability in the region: we have obtained two stable solutions for two different initial conditions at the same point in the phase plane (see ESI at ref. 51, Fig. 3). In contrast, the transition between DH(R) and PC(R) is smooth and happens under increased stretching for constant push (Fig. 4(g)).

### 3.3 Active polymerising drop

The polymerising drop, in addition to RP(L), LP(R), RP(R), and DB has two long and thin states (see length in Fig. 5(b)), the right-moving treadmilling state TM(R), which has a prominent hump at the rear followed by a small dip and a protrusion at the front, and the right-moving travelator, TL(R) which is flat almost everywhere, with the average height *h̄* < *h*_0_. The TM(R) shape is a solution to the 2D equation for a free drop driven by actin polymerisation for large enough self-advection velocity.^39^ Similar shapes with flat frontal protrusions have been observed experimentally for motile keratocytes.^16,19^ Both TM(R) and TL(R) drops have zero traction in the bulk Fig. 5(d). In ref. 39, where equal contact angles (rather than variable force) were imposed, the TM(R) shapes emerged when the dimensionless polymerisation speed 
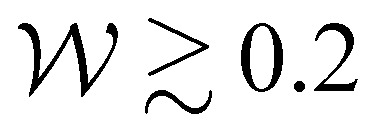
, showing that relatively small polymerisation speeds can generate stresses large enough to deform the drop. Again, there is large curvature at the humps, which take up 10–50% of the drop length, and the remaining drop is flat. At much lower polymerisation speed, 
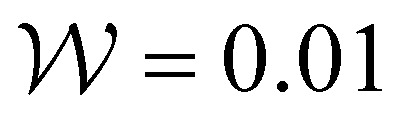
, (see ESI ref. 50), we find TM(R) and TL(R) shapes at large enough stretch (
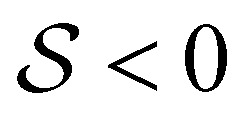
), which again shows that that stretching can amplify the effect of activity to deform the drop. Therefore, in the TM(R) and TL(R) drops, we see the competition between stress generated by polymerisation plus stretch, and surface tension: activity plus stretch wants to deform the drop, and surface tension wants the free surface to be flat.

Again, the symmetry about the push/pull axis is broken because the drop is motile at zero force, this time due to treadmilling self-advection, 
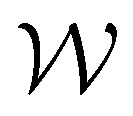
. The RP(L) region becomes smaller with self-advection, and is pushed to the top-left (push-left, squeeze), while the LP(R) region becomes larger with self-advection and invades the left half of the squeeze region. The RP(R) phase emerges as a thin strip between RP(L) and LP(R). DB for the polymerising drop occurs in the bottom-right (push-right, stretch) rather than in the bottom-left (push-left, stretch) as is the case for the contractile/extensile drop. The stretch region of the phase plane is populated by TM(R) and TL(R), as well as DB. The region containing TM(R) and TL(R) shares a phase boundary with both RP(R) and LP(R) determined by the second moment of *h* as a measure of the spread of the drop, and the distribution of substrate traction. The phase boundary is indicated in the schematic Fig. 2(c), as determined from the data in Fig. 5(b) and (d). Strikingly, the transition between RP(R)/LP(R) and DH(R)/PC(R) is sharp suggesting a first order transition. The behaviour of the second moment and the drop velocity across the phase boundary is shown in Fig. 5(e). There is a jump in both quantities going from RP(R)/LP(R) to TM(R)/TL(R). The change in drop shape induced by moving from LP(R) to TL(R) is shown Fig. 5(f). The drop shape changes continuously within the TM(R)/TL(R) region but changes sharply at the phase boundary. In contrast, the transition between TM(R) and TL(R) is smooth.

## 4 Discussion and parameter estimates

**Fig. 6:**
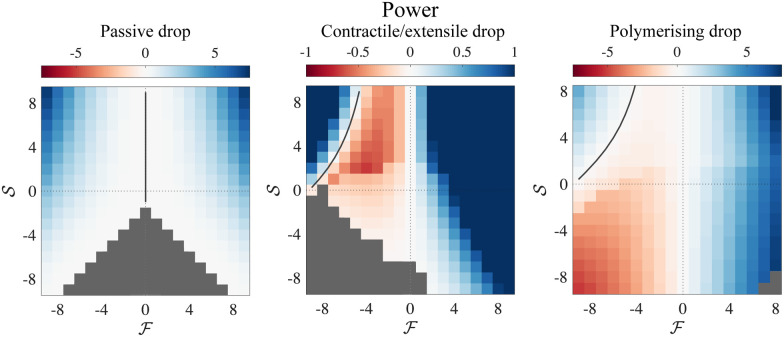
Left: Power (net force × velocity) for a passive drop. Middle: Power for an active contractile/extensile drop. Right: Power for an active polymerising drop.

The drastic changes in drop shape that we have observed are not associated with changes in direction of motion. Both the contractile/extensile drop and the polymerising drop remain as right-parabolas when their velocity is reversed at the RP(L)/RP(R) boundary. Stationary drop profiles are shown in Fig. 4(h). All drops we have considered here are fastest for strong push and strong squeeze, towards the top corners of the phase plane. The contractile/extensile drop slows down dramatically on entering the DH(R) region and continues to slow down as it enters the PC(R) region and approaches DB, however it does not stop before reaching DB. In contrast, the polymerising drop locally speeds up as it approaches the TM(R) region and continues to speed up as it enters the TL(R) region and approaches DB. We can also compute the active power generated by moving the drop against applied forces, showing that when the drop moves opposite the applied force, it acts as a motile engine (see Fig. 6).

We now estimate dimensionful parameter values for our model to provide biological context and make predictions. In our active simulations we looked at 
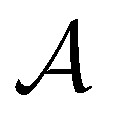
 between 0.3 and 1. Assuming a surface tension of 0.01 mN m^−1^,^52^ a length scale of 25 μm, and a drop inverse aspect ratio *ε* = 0.1, we can use (3) to calculate *α̃* to be between 0.02 pN (μm)^−2^ and 0.05 pN (μm)^−2^. This is comparable to a bottom-up estimate of activity parameter *α̃* from the force produced by myosin motors cross-linking and walking across actin filaments, which we treated as rods in water. We estimated the force produced by each rod to be the Stokes drag on a rod, *F*_rod_ ≈ 2π*Lηv*, where *L* is the length of an actin filament, which we take to be 1 μm, *η* is the viscosity of water, which is 8.9 × 10^−4^ Pa s, and *v* is the myosin walking speed, which we take to be 200 nm s^−1^.^53^ Using these numbers we estimate the force produced by a single actomyosin bundle to be 5 nN. We take the density of actin monomers in a cell to be 100 mg mL^−1^,^54^ the mass of an actin monomer to be 42 kDa,^55^ the volume of a cell to be 10^−15^ m^3^,^56^ and the number of actin monomers to per filament to be 370,^57^ which gives 4 × 10^5^ actin filaments per cell. Multiplying this number by the Stokes drag gives a total force of *F*_cell_ = 5 × 10^−10^ N. Since disordered actomyosin networks do not convert microscopic forces efficiently, we estimate the active force scale to be 0.01–0.1 *F*_cell_/(*L*_cell_^2^), where we have used *L*_cell_ ≈ 25 μm,^56^ so *α̃* ≈ 0.08–0.8 N m^−2^ = 0.08–0.8 pN (μm)^−2^.

Similarly, we can calculate *w̃*_0_ and *Ṽ via*eqn (3) using our assumed value of surface tension, and using the viscosity of a semi-flexible polymer gel, which we take to be 3 × 10^−1^ Pa s.^58^ We find *w̃*_0_ between 10 nm s^−1^ and 30 nm s^−1^, comparable to the range of polymerisation speeds, 7 nm s^−1^ to 170 nm s^−1^, for motile cells.^59^ Our model predicts cell speeds around 10 nm s^−1^. We use values of push and squeeze ranging from 0.04 pN (μm)^−2^ to 0.4 pN (μm)^−2^, and our model predicts a stall force of around 60 pN (assuming a contact area of 2 × 25 (μm)^2^).

## 5 Conclusion

In summary, we have studied the dynamics of a model cell (an active LC drop) on a flat surface under external forces applied at its two ends. Our phase diagrams in terms of the sum and differences of these forces and show how cell shape and motile behaviour is nonlinearly modulated by external forces. Our analysis has focused on two mechanisms of motility driven by contractile/extensile active stresses and actin polymerisation. Both mechanisms give rise to the same parabolic phases (RP(L), LP(R), RP(R)) under squeeze, and different phases under stretch: DH(R) and PC(R) for the contractile/extensile drop, and TM(R) and TL(R) for the polymerising drop.

These unexpectedly strong shape and motility changes under applied forces should help us understand similar changes in experiment. Epithelia change from columnar (tall) to squamous (flat) shapes under tension, and there is a tradeoff between active traction with the substrate and at the apical (top) surface. Similarly, in the mesenchymal–epithelial transition from flat or humped, strongly motile mesenchymal cells slow down and become taller when squeezed in by other cells.^1,5^ The changes in shape that we have observed here can be exploited to control behaviour of different cell phenotypes and tissue remodelling. In particular, the relation that we have derived between cell-substrate traction and forces, and between cell velocity and traction could be investigated by measuring the cell-substrate force using *e.g.* traction force microscopy and pushing/pulling the cell with a micropipette.

## Author contributions

TBL formulated the problem. AI, under the supervision of TBL and SH, derived the model equations, carried out the numerical calculations with the help of AL, and analysed the data. All authors contributed to writing the manuscript.

## Conflicts of interest

There are no conflicts to declare.

## Supplementary Material

SM-018-D2SM00934J-s001
