# Supplementary material for: The nonlinear motion of cells subject to external forces
Source: Soft Matter. 2022 Nov 10;18(47):9008–16. doi: 10.1039/d2sm00934j (PMC10141577; doi:10.1039/d2sm00934j)
Supplement: SM-018-D2SM00934J-s001 [file SM-018-D2SM00934J-s001.pdf]

Electronic Supplementary Material (ESI) for Soft Matter.
This journal is © The Royal Society of Chemistry 2022

Supplemental Material for The nonlinear motion of cells subject to external forces

Aondoyima Ioratim-Uba, Silke Henkes, Aurore Loisy, Tanniemola B. Liverpool
School of Mathematics, University of Bristol - Bristol BS8 1UG, UK

1 Deriving the height equation

In this section we will outline the derivation of $h'''(\frac{1}{3}h^3 + l_u h^2) + g(h) = \mathcal{V}h$ for the active contractile drop. The derivation for the active polymerising drop is almost identical except that there is no imposed winding and the the director angle therfore scales differently, activity α scales differently, and there is a non-zero advection velocity in the statement of mass conservation. We begin with the force balance equation in the lubrication approximation

$$\partial_{\tilde{x}}\tilde{p} - \tilde{f}_{\tilde{x}} = \eta\partial_{\tilde{z}}^2\tilde{u}_{\tilde{x}} - \tilde{\alpha}\partial_{\tilde{z}}(n_x n_z) = \partial_{\tilde{z}}\tilde{\sigma}_{\tilde{x}\tilde{z}}, \tag{1a}$$

$$\partial_{\tilde{z}}\tilde{p} = 0, \tag{1b}$$

where we have chosen

$$\tilde{f}_{\tilde{x}} = \tilde{f}_+\delta(\tilde{x} - \frac{\tilde{L}}{2}) + \tilde{f}_-\delta(\tilde{x} + \frac{\tilde{L}}{2}), \qquad \tilde{f}_{\tilde{z}} = 0. \tag{2}$$

For here, we will **omit the tildes**, but remember that the quantities in this section are **not** non-dimensionalised.

1.1 Mass conservation

We obtain the statement of mass conservation from the kinematic boundary boundary condition $\frac{D}{Dt}(h(x,t) - z) = 0$, where $\frac{D}{Dt} = \partial_t + (\mathbf{u} + w\mathbf{n}) \cdot \nabla$ is the material derivative. We then impose a travelling wave solution $h = h(x - Vt)$ on the kinematic condition, where V is the unknown constant drop velocity, to obtain this statement of mass conservation

$$\int_0^h (u_x + wn_x + V)\, dx = 0, \tag{3}$$

where we have made the transformation $x \leftarrow x - Vt$, so that now x is the centre of mass coordinate, and where where $\mathbf{u}$ is the fluid velocity inside the drop satisfying force balance and incompressibility, and $w\mathbf{n}$ describes the additional transport due to self-advection at speed w of active units whose orientations are characterised by the director $\mathbf{n}$. For the active contractile drop, which is the case we outline below, $w = 0$.

The strategy from here will be to first integrate (1a) at $\pm L/2$ to get boundary conditions on $p(\pm L/2)$ imposed by the Dirac deltas. We will then solve (1a) away from $x = \pm L/2$, and apply the derived boundary conditions at $\pm L/2$.

1.2 External forces impose boundary conditions on pressure

At $x = L/2$, we have

$$\int_{\frac{L}{2}-\Delta}^{\frac{L}{2}+\Delta} \partial_x p dx - f_+ \int_{\frac{L}{2}-\Delta}^{\frac{L}{2}+\Delta} \delta(x - \frac{L}{2}) dx - f_- \int_{\frac{L}{2}-\Delta}^{\frac{L}{2}+\Delta} \delta(x + \frac{L}{2}) dx = \int_{\frac{L}{2}-\Delta}^{\frac{L}{2}+\Delta} \partial_z \sigma_{xz} dx.$$

The last term on the LHS vanishes because of the definition of the delta function and the term on the RHS vanishes as we shrink the integration region ($\Delta \to 0^+$) because the integrand is continuous. The procedure is identical at $x = -L/2$. Thus we have

$$p(\frac{L}{2} + \Delta) - p(\frac{L}{2} - \Delta) - f_+ = 0, \qquad p(-\frac{L}{2} + \Delta) - p(-\frac{L}{2} - \Delta) - f_- = 0.$$

We model the system to have a uniform pressure $\pi_0 + \pi_{ref}$, where π_{ref} is an arbitrary reference pressure while π_0 is the uniform pressure that is present in the absence of activity α and external forces $F_\pm$, where the drop is stationary and symmetric. Because the external force is localised to $x = \pm L/2$ and we take the pressure to be uniform everywhere else outside the drop, it must be the case that $p(\frac{L}{2} + \Delta) = p(-\frac{L}{2} - \Delta) = \pi_0 + \pi_{ref}$. We also define

$$p(L/2) = \lim_{\Delta \to 0^+} p(L/2 - \Delta), \qquad p(-L/2) = \lim_{\Delta \to 0^+} p(-L/2 + \Delta),$$

resulting in the boundary conditions

$$p(\frac{L}{2}) = \pi_0 + \pi_{ref} - f_+, \tag{4a}$$

$$p(-\frac{L}{2}) = \pi_0 + \pi_{ref} + f_-. \tag{4b}$$

Solving equation (1a) is now equivalent to solving

$$\partial_x p = \partial_z \sigma_{xz} \tag{5}$$

with boundary conditions (4).

1.3 Pressure as a functional of drop height

The pressure $p(x)$ inside the drop can be related to the drop height $h(x)$ using the normal component of the free surface boundary condition (see main text)

$$\boldsymbol{m} \cdot \boldsymbol{\sigma} \cdot \boldsymbol{m} = \gamma\kappa - \pi_{ref}.$$

Using the scalings from the lubrication approximation, $\alpha \sim \epsilon^{-1}$, $p \sim \epsilon^{-2}$, $u \sim 1$, and $w \sim \epsilon$ (from incompressibility), $\sigma_{xx} = \sigma_{zz} \approx -p \sim \epsilon^{-2}$, $\sigma_{xz} \sim \epsilon^{-1}$, we can write the LHS of the above equation as

$$\begin{aligned}
\boldsymbol{m} \cdot \boldsymbol{\sigma} \cdot \boldsymbol{m} &= m_x \sigma_{xz} m_z + m_z \sigma_{xz} m_x + m_z \sigma_{zz} m_z + m_x \sigma_{xx} m_x \\
&\approx \frac{1}{1 + (h')^2} (-p(h')^2 - p - 2h'\sigma_{xz}) \\
&= -p - 2\frac{h'\sigma_{xz}}{1 + (h')^2} \\
&\approx -p.
\end{aligned}$$

Approximating κ to leading order, we have

$$\kappa = \frac{h''}{(1+(h')^2)^{\frac{3}{2}}} \approx h''.$$

Thus

$$p(x) = -\gamma h'' + \pi_{ref}. \tag{6}$$

1.4 Height equation

To get and ODE for $h(x)$ we write u_x in terms of h and $\partial_x p$, substitute this into mass conservation (3) and rearrange to get $\partial_x p$ in terms of h, i.e. $\partial_x p = f(h)$. We then integrate $\partial_x p = f(h)$ and use the boundary conditions (4) to calculate the drop velocity V, this turns out to be a functional of h, i.e $V \sim \int_{-L/2}^{L/2} f(h)\, dx$. After this, the ODE for h is given by substituting (6) into $\partial_x p = f(h)$.

To get u_x in terms of h and $\partial_x p$, we integrate (5), where $\sigma_{xz} = \eta \partial_z u_x - \alpha n_x n_z$ at leading order, twice with respect to z, using the partial slip boundary condition at the substrate and the tangential component of the free surface boundary condition (see main text) (which can be re-written as a condition on $\partial_z u_x(z = h)$). We then have

$$u_x = \frac{\alpha h}{4\pi\omega\eta}\left(1 - \cos\frac{2\omega\pi z}{h}\right) + \frac{\partial_x p}{\eta}\left(\frac{z^2}{2} - h(z + l_u)\right), \tag{7}$$

where we have used $\theta = \omega\pi z/h$.

Substituting (7) into (3) and isolating the pressure gradient yields

$$\partial_x p = \frac{\eta(\tilde{\alpha} h - V)}{\frac{1}{3}h^2 + l_u h}, \tag{8}$$

where $\tilde{\alpha} = \frac{\alpha}{4\pi\omega\eta}$. From here the strategy will be to integrate (8) and apply the boundary conditions (4) in order to determine the integration constant and the unknown drop velocity V. We find that the drop velocity is given by

$$\eta V = \frac{\int_{-L/2}^{L/2} \frac{\eta\tilde{\alpha}}{\frac{1}{3}h + l_u}\, dx + (f_+ + f_-)}{\int_{-L/2}^{L/2} \frac{1}{\frac{1}{3}h^2 + l_u h}\, dx}. \tag{9}$$

As a sanity check, we see that the second term in the numerator vanishes when the forces are equal and opposite, which means that the drop velocity in unchanged by the forces when they cancel each other out. This is good. The drop velocity also vanishes when the activity α and both forces vanish, which is good. From here we can substitute into (6) (8) to obtain

$$\frac{\gamma h'''}{\eta}\left(\frac{1}{3}h^3 + l_u h^2\right) + \frac{\alpha h^2}{4\pi\omega\eta} = Vh, \tag{10}$$

where the drop velocity V is given by (9). For the active polymerising drop, the same procedure applies, except we have $\omega = 0$, $\theta \sim \epsilon$, $\alpha \sim \epsilon^{-2}$, and the advection velocity $w > 0$. We set $\alpha = 0$ in the final result to get the equation in the main text.

2 Additional phase diagrams

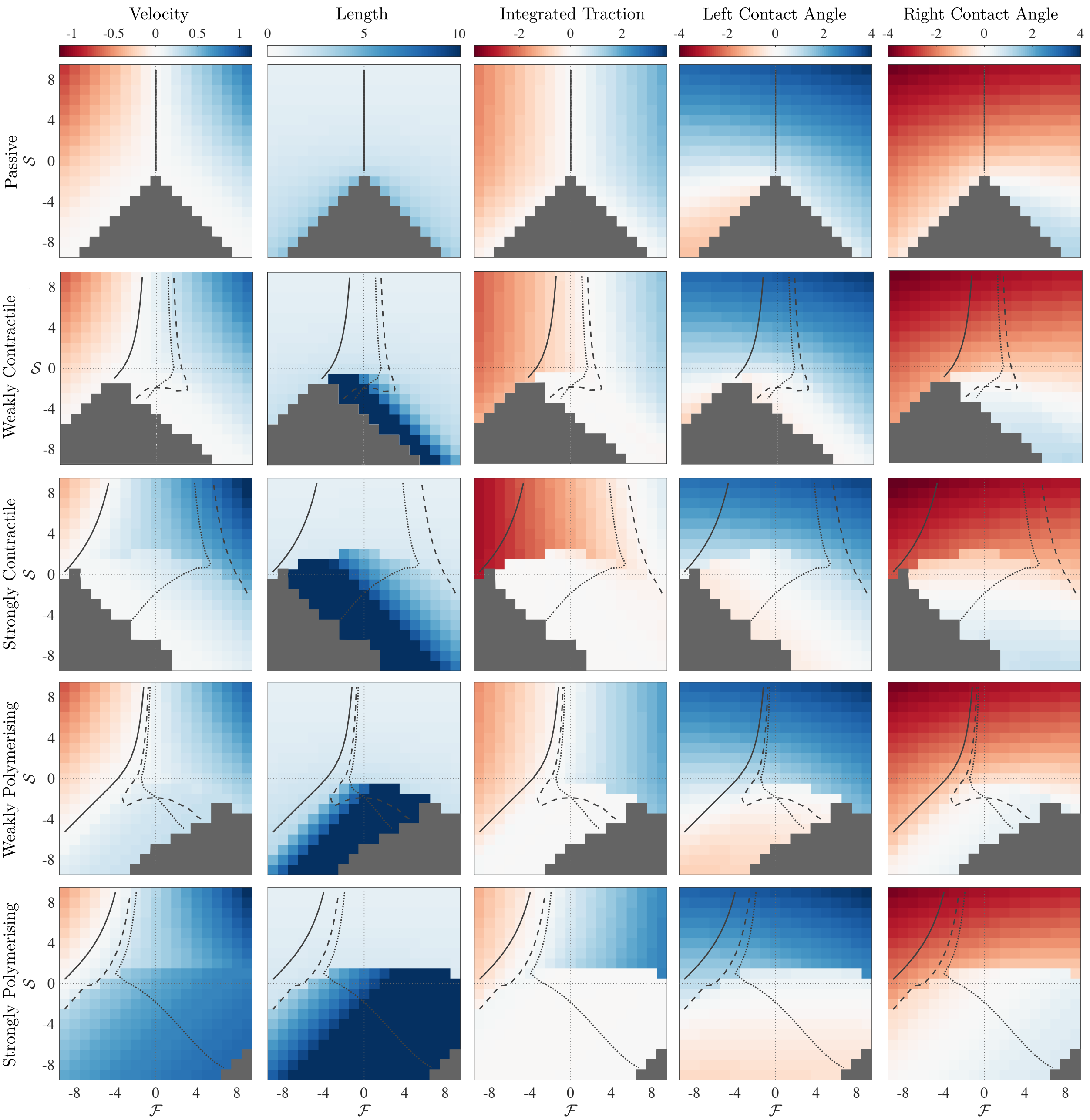

Figure 1: First row: passive drop ($\mathcal{A} = 0$), second row: weakly contractile ($\mathcal{A} = 0.3$), third row: strongly contractile ($\mathcal{A} = 1$), fourth row: weakly polymerising ($\mathcal{W} = 0.3$), fifth row: strongly polymerising ($\mathcal{W} = 1$). First column: drop velocity. Second column: drop length. Third column: raction between the drop and the substrate integrated over 70% of the drop length. Fourth column: left contact angle $\phi_- = h'(-L/2)$, fifth column: right contact angle $\phi_+ = h'(L/2)$. The solid line in each plot is an isoline corresponding to zero velocity, the dashed line is an isoline corresponding to the the 1st moment $\mu_1 = 0$, and the dotted line is an isoline corresponding to the drop having equal contact angles.

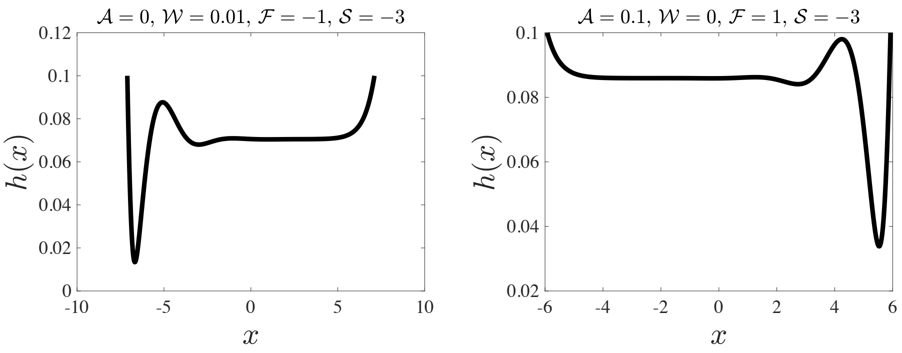

Figure 2: Left: profile for a drop with small polymerisation speed under stretch. Right: profile for a drop with small contractile stress under stretch

3 Solving the height equation with the Crank-Nicholson method

The equation that is fed into the Crank-Nicholson algorithm is

$$\partial_t h + \partial_x(-\mathcal{V}h + h'''(\frac{1}{3}h^3 + l_u h^2) + g(h)) = 0, \tag{11}$$

where $g(h) = \mathcal{A}h^2$ for the active contractile drop, $g(h) = \mathcal{W}l_w(1 - \exp(-h/l_w))$ for the active polymerising drop, and the drop velocity $\mathcal{V}$ is defined in the main text. Equation (11) is subject to the constraints

$$\int_{-L/2}^{L/2} h(x)\,dx = \Omega, \qquad h(\pm L/2) = h_0, \tag{12}$$

where Ω is the dimensionless drop volume, and

$$h''(-L/2) + \pi_0 + f_- = 0, \qquad h''(L/2) + \pi_0 - f_+ = 0, \tag{13}$$

with

$$\pi_0 = \frac{2\phi}{-\frac{3h_0}{\phi} + \sqrt{\frac{9h_0^2}{\phi^2} + \frac{6\Omega}{\phi}}},$$

where ϕ is the re-scaled contact angle.

Equation (11) is of the form $\partial_t h = I$, where I represents the second term on the LHS of (11). The Crank-Nicholson scheme advances in time according to

$$\frac{h_i^{n+1}}{\Delta t} - \frac{1}{2}I_i(\mathbf{X}^{n+1}) = \frac{h_i^n}{\Delta t} + \frac{1}{2}I_i(\mathbf{X}^n), \tag{14}$$

where the subscripts refer to the spatial discretisation and the superscripts refer to the time discretisation. Note that in the second term on the LHS, $I_i(\mathbf{X}^{n+1})$, means I evaluated at spatial point i at time-step $n+1$.

3.1 Spatial discretisation

For the discretisation we use the substitution $x = Ly$, with L being the drop length, and discretise the domain $y\epsilon[-0.5, 0.5]$ with uniform grid spacing Δy. The stiff term is discretised as follows

$$\partial_x\left(h''\left(\frac{1}{3}h^3 + l_u h^2\right)\right) \to \frac{\left[(\frac{1}{3}h_i^3 + l_u h_i^2) + (\frac{1}{3}h_{i+1}^3 + l_u h_{i+1}^2)\right](h_{i+2} - 3h_{i+1} + 3h_i - h_{i-1})}{2L^4\Delta y^4} - \frac{\left[(\frac{1}{3}h_{i-1}^3 + l_u h_{i-1}^2) + (\frac{1}{3}h_i^3 + l_u h_i^2)\right](h_{i+1} - 3h_i + 3h_{i-1} - h_{i-2})}{2L^4\Delta y^4},$$

and all other terms are discretised as

$$\partial_x G(h) \to \frac{G(h_{i+1}) - G(h_{i-1})}{2L\Delta y}.$$

3.2 Algorithm and boundary conditions

Equation (14) leads to a set of nonlinear algebraic equations for $\{h_i^{n+1}\}$, where $\{h_i^n\}$ are known, which is solved using the Matlab fsolve algorithm. For N spatial grid points, define $\mathbf{X}^{n+1} = (h_1, ..., h_N)^{n+1}$. The algorithm solves $\mathbf{F}(\mathbf{X}^{n+1}) = \mathbf{0}$ with

$$\mathbf{F}(\mathbf{X}^{n+1}) = \frac{\mathbf{X}^{n+1}}{\Delta t} - \frac{1}{2}\mathbf{I}(\mathbf{X}^{n+1}) - \frac{\mathbf{X}^n}{\Delta t} - \frac{1}{2}\mathbf{I}(\mathbf{X}^n) \qquad i = 3, ..., N-2, \tag{15}$$

where $\mathbf{I} = (I_1, ..., I_N)$, with I_i being the spatial differential operator evaluated at spatial point i. The boundary conditions are implemented using grid points $1, 2, N-1, N$. The condition on the drop height at $\pm L/2$ is implemented as

$$F_1 = h_1^{n+1} - h_0 \tag{16a}$$

$$F_N = h_N^{n+1} - h_0. \tag{16b}$$

The boundary conditions on the second derivative (13) are implemented using finite difference coefficients to approximate the second derivative:

$$F_2 = \frac{2h_1^{n+1} - 5h_2^{n+1} + 4h_3^{n+1} - h_4^{n+1}}{\Delta y^2} + L^2\left(\frac{\pi_0 + f_-}{\tilde{C}}\right), \tag{17a}$$

$$F_{N-1} = \frac{2h_N^{n+1} - 5h_{N-1}^{n+1} + 4h_{N-2}^{n+1} - h_{N-3}^{n+1}}{\Delta y^2} + L^2\left(\frac{\pi_0 - f_+}{\tilde{C}}\right). \tag{17b}$$

Once equation (11) is discretised, we use a nonlinear solver (MATLAB's "fsolve") for the simultaneous equations $\mathbf{F}(\mathbf{X}^{n+1}) = \mathbf{0}$, with the components of $\mathbf{F}$ given by (15), (16), and (17). We also calculate the Jacobian $\partial F_i/\partial_{h_j}$ explicitly and supply it to the nonlinear solver. The algorithm begins with a user set initial condition $\mathbf{X}^1$ which is chosen to satisfy the boundary conditions on drop height but not necessarily the boundary conditions on the second derivative. The drop velocity $\mathcal{V}$ as well as the drop length L are calculated iteratively starting from the initial condition (the drop length is derived from the constraint that the drop has a constant volume) and plugged into the Crank-Nicholson evolution equation which is solved for $\mathbf{X}^2$. This process is repeated until steady state is reached i.e. the difference between $\mathbf{X}^{n+1}$ and $\mathbf{X}^n$ is less than some tolerance.

4 Asymptoics for the active contractile drop

We expand the equation

$$h'''(\frac{1}{3}h^3 + l_u h^2) + \mathcal{A}h^2 = \left(\frac{\mathcal{A}I_1 + (f_+ + f_-)}{I_2}\right)h, \tag{18}$$

where $I_1 = \int_{-L/2}^{L/2}(\frac{1}{3}h + l_u)^{-1}\,dx$ and $I_2 = \int_{-L/2}^{L/2}(\frac{1}{3}h^2 + l_u h)^{-1}\,dx$, for small activity and small forces.

4.1 Small forces and small activity

We consider small perturbations to a symmetric passive drop by expanding $h(x)$ to linear order in $f_\pm$ and $\mathcal{A}$ around the passive solution, obtained by setting $f_+ = f_- = 0$ and $\mathcal{A} = 0$ in (18) and either imposing $h''(x) = \pi_0$ or $h'(\frac{\pm L}{2}) = \mp\phi$ along with $h(\frac{\pm L}{2}) = h_0$:

$$h_p = -\frac{\phi L}{4}\left(\frac{4x^2}{L^2} - 1\right) + h_0. \tag{19}$$

We also expand L to linear order in $f_\pm$ and $\mathcal{A}$:

$$L = L_p - f_+ L_+ - f_- L_- + \mathcal{A}L_\alpha + \cdots \tag{20}$$

which leads to

$$\begin{aligned} h(y) =& H_0 + f_+\left(\frac{\phi L_+}{4}(y^2-1) - h_+\right) + f_-\left(\frac{\phi L_-}{4}(y^2-1) - h_-\right) \\ &+ \mathcal{A}\left(h_\alpha - \frac{\phi L_\alpha}{4}(y^2-1)\right) + \cdots \end{aligned} \tag{21}$$

where $y = 2x/L$, $H_0 = -\frac{\phi L_p}{4}(y^2 - 1) + h_0$, and L_p is the length of the passive drop given by $L_p = \sqrt{\frac{6\Omega}{\phi} + \frac{9h_0^2}{\phi^2}} - \frac{3h_0}{\phi}$ for a drop of volume Ω. Substituting (21) into (18) and keeping terms only to linear order yields three differential equations at $\mathcal{O}(f_+)$, $\mathcal{O}(f_-)$, and $\mathcal{O}(\mathcal{A})$ that can be integrated for $h_\pm$ and h_α. At $\mathcal{O}(f_+)$ we have

$$h_+'''(x)(\frac{1}{3}H_0^2 + l_u H_0) = -\frac{1}{I_2}, \qquad h_+(\pm 1) = 0, \qquad h''(1) = -\frac{L_p^2}{4} - \frac{\phi L_+}{2}. \tag{22}$$

For consistency, we must have also $h_+''(-1) = -\phi L_+/2$ but we cannot impose this on the equation, as there are already three boundary conditions. Fortunately, it falls out automatically because the integrals I_1 and I_2 encode both boundary conditions on h''. At $\mathcal{O}(f_-)$ we have

$$h_-'''(x)(\frac{1}{3}H_0^2 + l_u H_0) = -\frac{1}{I_2}, \qquad h_-(\pm 1) = 0, \qquad h_-''(-1) = \frac{L_p^2}{4} - \frac{\phi L_-}{2}. \tag{23}$$

Again, for consistency, we must have $h''_-(1) = -\phi L_-/2$, which again falls out automatically. At $\mathcal{O}(\mathcal{A})$ we have

$$h'''_\alpha(x)(\frac{1}{3}H_0^2 + l_u H_0) = \frac{I_1}{I_2} - H_0, \qquad h_\alpha(\pm 1) = 0, \qquad h''_\alpha(1) = -\frac{\phi L_\alpha}{2}. \tag{24}$$

For consistency we must have $h''_\alpha(-1) = -\phi L_\alpha/2$, which falls out automatically. To perform the integration, we use the variable $y = \dfrac{2x}{L}$. It is useful to define the following function

$$\begin{aligned} G_f(y) =& \left(1 + \frac{y}{\sqrt{\Gamma}}\right)^2 \log\left(1 + \frac{y}{\sqrt{\Gamma}}\right) - \left(1 - \frac{y}{\sqrt{\Gamma}}\right)^2 \log\left(1 - \frac{y}{\sqrt{\Gamma}}\right) \\ &+ \frac{1}{\beta\phi}\left[\left(\beta - \frac{\phi y}{\sqrt{\Gamma}}\right)^2 \log\left(\beta - \frac{\phi y}{\sqrt{\Gamma}}\right) - \left(\beta + \frac{\phi y}{\sqrt{\Gamma}}\right)^2 \log\left(\beta + \frac{\phi y}{\sqrt{\Gamma}}\right)\right], \end{aligned}$$

where

$$\Gamma = 1 + \frac{4h_0}{\phi L_p},$$

and

$$\beta = \sqrt{\frac{\Gamma + \dfrac{12 l_u}{\phi L_p}}{\Gamma}}.$$

We also need

$$G_\alpha(y) = \frac{\phi}{4\beta}\left[\left(\frac{\beta}{\phi} + \frac{y}{\sqrt{\Gamma}}\right)^2 \log\left(\frac{\beta}{\phi} + \frac{y}{\sqrt{\Gamma}}\right) - \left(\frac{\beta}{\phi} - \frac{y}{\sqrt{\Gamma}}\right)^2 \log\left(\frac{\beta}{\phi} - \frac{y}{\sqrt{\Gamma}}\right)\right].$$

In terms of these functions, the leading order contributions to the drop height are

$$h_+(y) = \frac{-L_p^2(1 + \dfrac{4h_0}{\phi L_p})}{8G''_f(1)}\left[G_f(y) + \frac{1}{2}G''_f(1))\left(1 + \frac{4\phi}{L}{}_+ L_p^2\right)y^2 - \sqrt{1 + \frac{4h_0}{\phi L_p}}G_f(1)y - \frac{G''_f(1)(1 + 4\phi L_+/L_p^2)}{2(1 + 4h_0/\phi L_p)}\right], \tag{25a}$$

$$h_-(y) = \frac{-L_p^2(1 + \dfrac{4h_0}{\phi L_p})}{8G''_f(1)}\left[G_f(y) - \frac{1}{2}G''_f(1)\left(1 - \frac{4\phi L_-}{L_p^2}\right)y^2 - \sqrt{1 + \frac{4h_0}{\phi L_p}}G_f(1)y + \frac{G''_f(1)(1 - 4\phi L_-/L_p^2)}{2(1 + 4h_0/\phi L_p)}\right], \tag{25b}$$

$$h_\alpha(y) = \frac{3L_p^2\sqrt{1 + \dfrac{4h_0}{\phi L_p}}}{2\phi}\left[\frac{G''_\alpha(1)}{G''_f(1)}G_f(y) - G_\alpha(y) + \sqrt{1 + \frac{4h_0}{\phi L_p}}\left(G_\alpha(1) - \frac{G''_\alpha(1)}{G''_f(1)}G_f(1)\right)y\right], \tag{25c}$$

where

$$L_+ = \frac{-L_p^3}{24}\left(\frac{\phi L_p}{2} + h_0\right)^{-1}, \tag{26a}$$

$$L_- = \frac{L_p^3}{24}\left(\frac{\phi L_p}{2} + h_0\right)^{-1}, \tag{26b}$$

$$L_\alpha = 0. \tag{26c}$$

5 Hints of bi-stability

Both the active contractile drop and active polymerising drop can produce different steady state drop profiles in the iterative numerical scheme, starting from different height profiles with the same activity and applied forces. This is shown in figure 5 for the active contractile drop at the RP(R)/DH(R) boundary. For the active polymerising drop, this behaviour occurs at the LP(R)/TM(R) boundary.

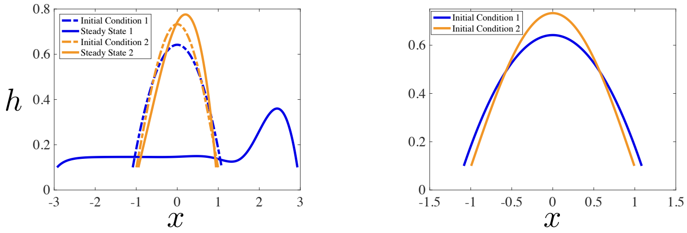

Figure 3: Left: Initial height profiles (dashed lines) and steady state profiles (solid lines) for Right: Only the initial height profiles. Here $\mathcal{A} = 1$, $\mathcal{F} = -2$, and $\mathcal{S} = 2$.
